# Practical implications of naloxone knowledge among suburban people who use opioids

**DOI:** 10.1186/s12954-021-00466-8

**Published:** 2021-04-28

**Authors:** Kristin E. Schneider, Glenna J. Urquhart, Saba Rouhani, Ju Nyeong Park, Miles Morris, Sean T. Allen, Susan G. Sherman

**Affiliations:** 1 Department of Mental Health, Johns Hopkins Bloomberg School of Public Health, 624 N. Broadway, HH886, Baltimore, MD 21205 USA; 2 Department of Health, Behavior and Society, Johns Hopkins Bloomberg School of Public Health, 624 N. Broadway, Baltimore, MD 21205 USA

**Keywords:** Naloxone, Overdose, Opioids, Naloxone access

## Abstract

**Background:**

Naloxone distribution programs have been a cornerstone of the public health response to the overdose crisis in the USA. Yet people who use opioids (PWUO) continue to face a number of barriers accessing naloxone, including not knowing where it is available.

**Methods:**

We used data from 173 PWUO from Anne Arundel County, Maryland, which is located between Baltimore City and Washington, DC. We assessed the prevalence of recently (past 6 months) receiving naloxone and currently having naloxone, the type(s) of the naloxone kits received, and the perceived ease/difficultly of accessing naloxone. We also assessed participants knowledge of where naloxone was available in the community.

**Results:**

One third (35.7%) of participants had recently received naloxone. Most who had received naloxone received two doses (72.1%), nasal naloxone (86.9%), and education about naloxone use (72.1%). Most currently had naloxone in their possession (either on their person or at home; 78.7%). One third (34.4%) believed naloxone was difficult to obtain in their community. Only half (56.7%) knew of multiple locations where they could get naloxone. The health department was the most commonly identified naloxone source (58.0%). Identifying multiple sources of naloxone was associated with being more likely to perceive that naloxone is easy to access.

**Discussion:**

Our results suggest that additional public health efforts are needed to make PWUO aware of the range of sources of naloxone in their communities in order to ensure easy and continued naloxone access to PWUO.

## Introduction

As the overdose epidemic has ravaged communities across the USA, naloxone distribution has been a core strategy to reduce opioid overdose morbidity and mortality [1, 2]. Naloxone is an opioid antagonist medication that reverses opioid overdoses [3, 4]. Naloxone is available as a nasal spray and as an intramuscular injectable. Naloxone distribution efforts range from co-prescribing naloxone with opioid prescriptions, to community outreach programs training lay persons in overdose response, to standing orders which allow pharmacists to dispense naloxone without individual prescriptions [2, 5]. States have designated service providers, like homeless shelters, syringe services programs (SSPs), and fire departments, as overdose response programs, allowing them to distribute naloxone [6].

The proliferation of illicitly manufactured fentanyl and its analogues in the drug supply has presented challenges for overdose reversal through naloxone [7]. Fentanyl-associated overdoses may require more naloxone than heroin to reverse and more commonly re-emerge after apparent reversal [8]. In the fentanyl era, it is more important than ever that all individuals at risk for experiencing or witnessing an overdose have ample and easy access to naloxone in order to prevent overdose fatalities.

People who use opioids (PWUO) experience structural barriers to naloxone access, including poverty, homelessness, and stigma [9, 10], which are exacerbated by insufficient naloxone availability and lack of familiarity of where to access. Structural barriers can act to prevent naloxone access in a variety of ways, such as experiences of stigma making a person unwilling to access a particular program or service or poverty making a person unable to afford pharmacy fees. While knowledge alone is not sufficient to ensure naloxone possession [11], it is a necessary prerequisite for PWUO to seek out naloxone and to replace doses. We explored naloxone experiences among a sample of primarily suburban PWUOs and assessed their knowledge of where to get naloxone in their community.

## Methods

We used data from the Peer harm Reduction of Maryland Outreach Tiered Evaluation (PROMOTE), a mixed-methods, cross-sectional study in Anne Arundel County, Maryland. Anne Arundel County is situated between Baltimore City, MD, and Washington D.C., and contains the capital of Maryland, Annapolis. Anne Arundel County is formally considered to be part of Baltimore City’s metropolitan statistical area but is best characterized as suburban due to its substantially lower population density (1296/mile in Anne Arundel, 7672/mile in Baltimore City) [12, 13]. Anne Arundel is broadly constituted by a variety of small towns and communities separated by undeveloped land (i.e., forests and rivers). There is little walkability between towns and limited public transportation between communities. Homelessness is low in the county, with a 2019 estimate of 0.22% prevalence [14].

Participants were 18 or older and used non-prescription opioids in the past 6 months. A total of 173 PWUO were recruited from November 2019 to March 2020; data collection was stopped prematurely due to the COVID-19 pandemic. Recruitment locations were identified via geospatial analyses of drug-related arrest data and fatal/non-fatal overdose data provided by county and city police departments. Using these data, we developed heat maps in ArcMap 10.4.1 and identified 7 geographical locations with possible drug-related activities. We then extracted the time signatures associated with arrests in each area to develop a sampling frame that consisted of venue-day-time units (geographical location, day of the week, four-hour periods), which study staff drove the study van to using a predetermined schedule to recruit.

After providing informed consent, participants completed a 30-min audio computer-assisted self-interview. Respondents were compensated with a $20 VISA gift card. We removed two participants missing on all the naloxone variables (analytic *n* = 171). This study was approved by the Johns Hopkins Bloomberg School of Public Health Institutional Review Board.

Participants reported their age, gender (man, woman, transgender/non-binary), race (Black, white, other), if they were currently homeless (yes/no), and if they had ever injected drugs (yes/no). Participants were asked a series of naloxone-related questions. First, we asked if participants had recently (past 6 months) received naloxone for later use in the past (yes/no). Among those who had received naloxone, we asked if they currently had naloxone (yes/no), what type of naloxone they received (nasal, intramuscular, autoinjector; not mutually exclusive), the number of doses they received (1, 2, 3, 4 or more), if they had received education the last time they got naloxone, and if they had ever administered naloxone to someone overdosing (yes/no). Among those who had administered naloxone, we asked if the overdose required more than two doses to reverse (yes/no). We also asked all participants how easy or difficult it was to get naloxone in their community (very easy, somewhat easy, somewhat difficult, very difficult; an indicator of very/somewhat easy vs. very/somewhat difficult was created). Finally, we provided participants with a list of naloxone sources and asked them to indicate which, to their knowledge, were places where they could obtain naloxone. The list of naloxone sources included: health department, syringe/needle exchange, doctor’s office, hospital/emergency room, drug treatment/methadone clinic, pharmacy, police station, fire department, homeless shelter, and other.

We conducted a descriptive analysis of naloxone experiences, perceived ease of access, and knowledge of where to get naloxone using Stata 14 [15]. We estimated the prevalence of each naloxone variable in our sample. We then tested if perceived ease of access (binary indictor) was associated with previously receiving naloxone, receiving naloxone education, and the number of sources of naloxone participants could identify using Pearson’s χ^2^ tests.

## Results

The average age of participants was 41.5 years (12.1 SD). About two-thirds identified as men (65.3%) and one third were women (33.0%). The majority identified as Black (67.1%) followed by White (21.6%) or another race (11.4%). Close to two-thirds (60.1%) were currently experiencing homelessness. One third (31.4%) had ever injected drugs and 13.5% injected daily or more.

About one third (35.7%) of participants had recently received naloxone (Table 1). Among those who had received it, most currently had naloxone (78.7%). Naloxone kits received by participants primarily contained nasal naloxone (87%), and they typically had two doses (72%). Most who received naloxone also received education about it (72.1%). About half who had received naloxone used it to reverse an overdose (56.9%), and most of those who administered naloxone reported that it took more than two doses to reverse the overdose (79.3%).

**Table 1:** Previous experiences with naloxone in a sample of suburban people who use opioids

	*N* (%)
Received naloxone, past 6 months (*n* = 171)	
Yes	61 (35.7)
No	110 (64.3)
Currently have naloxone (*n* = 61)	
Yes	48 (78.7)
No	13 (21.3)
Type of THN received (*n* = 61)	
Nasal	53 (86.9)
Intramuscular	10 (16.4)
Auto injector	7 (11.5)
Number of doses received (*n* = 61)	
1	7 (11.5)
2	44 (72.1)
3	7 (11.5)
4+	3 (4.9)
Received education (*n* = 61)	
Yes	44 (72.1)
No	17 (27.9)
Ever administered naloxone for an overdose (*n* = 61)	
Yes	29 (56.9)
No	22 (43.1)
Needed more than two doses to reverse an overdose (*n* = 29)	
Yes	23 (79.3)
No	6 (20.7)

About one third thought naloxone was very easy to access (29.4%), another third thought it was somewhat easy (36.2%) to access in their community, and the remaining third thought it was somewhat (18.4%) or very (16.0%) difficult to access (Table 2). When asked about places where they could obtain naloxone, more than half (56.7%) were able to identify two or more sources, one third (31.6%) could identify one source, and few (11.7%) could not identify any source. The most commonly identified naloxone source was the health department (58.0%), followed by drug treatment facilities (43.8%), and hospitals/emergency rooms (32.7%). One quarter of participants were aware naloxone was available at pharmacies (26.5%) or the syringe program (24.1%). Police stations (9.9%) and “other” sources (4.9%) were the least commonly identified.

**Table 2:** Ease of access and knowledge of naloxone sources in a sample of suburban people who use opioids

	*N* (%)
Perceived ease of naloxone access (*n* = 163)	
Very difficult	26 (16.0)
Somewhat difficult	30 (18.4)
Somewhat easy	59 (36.2)
Very easy	48 (29.4)
Number of naloxone sources identified (*n* = 171)	
0	20 (11.7)
1	54 (31.6)
2 or more	97 (56.7)
Knowledge of naloxone availability from different sources (*n* = 171)	
Harm reduction sources	
Health department	94 (58.0)
Syringe/needle exchange	39 (24.1)
Healthcare sources	
Drug treatment/methadone clinic	71 (43.8)
Hospital/emergency room	53 (32.7)
Pharmacy	43 (26.5)
Doctor’s office	33 (20.4)
Other public service sources	
Fire station	39 (24.1)
Homeless shelter	33 (20.4)
Police station	16 (9.9)
Other	8 (4.9)

Participants who received naloxone were more likely to believe naloxone was easy to access than those who had not received it (76.7% vs 50.2%; *p* = 0.024; data not displayed). Among those who had received naloxone, receiving education was not associated with perceived ease of access (*p* = 0.484). Being able to identify two or more naloxone sources was associated with higher perceived ease of naloxone access (77.1%) compared to identifying only one (48.1%) or zero (57.1%) sources (*p* = 0.001).

## Discussion

We explored experiences with naloxone and knowledge of where naloxone could be accessed in a sample of suburban PWUO in Maryland. We found that one third of participants had recently received naloxone, among whom half had used it to respond to an overdose. About one third perceived that naloxone was difficult to access in their community. Most participants could identify at least one source of naloxone, and about half could identify multiple sources. Our results have important practical implications for improving naloxone coverage for PWUO.

Most who had responded to an overdose needed more than two doses to reverse the overdose. This contrasts with the observation that most received two doses the last time they accessed naloxone. Two dose naloxone kits are typical in many settings, though some programs offer more doses [16–18]. In the era of illicitly manufactured fentanyl, overdoses that require multiple doses of naloxone to reverse have become common [19]. This implies that naloxone distribution programs need to implement more liberal policies, where kits include more doses of naloxone to ensure that PWUO are equipped with sufficient supplies to reverse any overdose. It is important to acknowledge that programs experience salient barriers to expanding naloxone distribution, namely limited resources to purchase additional naloxone and potential shortages that need to be addressed through expanded funding to further the goal of reducing overdose fatalities.

Our study provides insight into knowledge about different sources of naloxone in community among PWUO, an underdeveloped area of the literature. In this sample, the health department and drug treatment programs were the two most commonly identified sources of naloxone. Only one quarter of participants knew that naloxone was available at a pharmacy. Low awareness that naloxone is available at pharmacies contrasts with the public health focus on promoting pharmacies as a major access point for naloxone [5, 20, 21]. Many cities and states, including Maryland, have standing orders for naloxone, meaning that anyone can access it at a pharmacy without a personal prescription if they are able to pay for it [5, 22]. This finding highlights a disconnect between policy efforts and actual education provided to PWUO. Without sufficient education efforts to make those at risk for witnessing or experiencing an overdose of policy changes to improve pharmacy-based naloxone access, these policies cannot have a meaningful health impact. Locales that aim to use pharmacies as a major source of naloxone must thoroughly communicate with at risk groups, like PWUO, about pharmacies as an option for obtaining naloxone and what the associated costs for filling a naloxone prescription may be. Further, governments need to take action to ensure that pharmacies provide naloxone for free to PWUO, as the high levels of poverty and other structural vulnerability in this population make even small costs as significant barrier.

One third of participants believed that it was difficult to access naloxone in their community, an additional third only believed naloxone was somewhat easy to access. This is likely due, in part, to the lack of SSPs in the county, meaning that individuals need to travel to nearby cities to access such programs. Previously accessing naloxone and being able to identify multiple sources were both associated with being more likely to perceive easy access. As knowledge of multiple naloxone sources was associated with easier perceived access, media campaigns to increase the awareness of different sources are warranted. Such campaigns should be specifically designed to reach PWUO who may be experiencing homelessness or other forms of marginalization, including through advertisements at programs and locations frequented by this population (i.e., homeless shelters and meal services). Education provided when naloxone is distributed should be modified to specifically include information about multiple naloxone sources in the community. It is important that individuals are aware of many different sources of naloxone to ensure ongoing and easy access. For example, PWUO may not be comfortable accessing naloxone at a pharmacy or from a medical provider, due to concerns about stigma or identifying themselves as people who use drugs, so for these individuals the health department and SSPs may be essential. Alternatively, those who do not inject drugs or who may not be able to easily reach the health department, due to a lack of transportation or limited mobility, may only be able to access naloxone from a local pharmacy. PWUO but who do not inject drugs may perceive that they have a low risk of overdose [23] and may therefore place a lower priority on obtaining naloxone, especially if they believe it is difficult to access. Increasing awareness of different channels to access naloxone is an essential step forward in improving coverage and prevention overdose mortality.

Findings should be viewed in light of study limitations. Our sample size is modest, limiting our ability to explore additional correlates. Our findings may only generalize to settings with similar policy environments. Maryland has enacted multiple policies and programs to ensure easy naloxone access including standing orders and dropping requirements for training to purchase naloxone, in addition to large-scale efforts to distribute naloxone to lay persons. In settings without naloxone standing orders or other programs to make naloxone readily available, perceived ease of access and knowledge of where to get naloxone are likely much lower than in our sample.

This study informs two key public health recommendations. Naloxone distribution programs should provide more doses to individuals to combat the effects of fentanyl. Local health departments, especially in suburban settings, need to improve education efforts to make PWUO aware of different sources of naloxone in the community. Changes are needed to achieve more complete naloxone coverage among suburban PWUO and curb the devastating impact of overdose fatalities in the USA.

## Data Availability

The data analyzed in this study are not publicly available.
